# Microstructure Characteristics and Its Effect on the Fracture in the Triple Junction Region of Friction Stir Welded Mg Alloys Subjected to Tension

**DOI:** 10.3390/ma13173672

**Published:** 2020-08-20

**Authors:** Guodong Liu, Qunying Yang, Yongshan Cheng

**Affiliations:** 1 College of Materials Science and Engineering, Yangtze Normal University, Chongqing 408100, China; yqy412829912@126.com; 2 School of Electronic Information Engineering, Yangtze Normal University, Chongqing 408100, China; 17347846030@163.com

**Keywords:** twin band, fracture, friction stir weld, magnesium alloy

## Abstract

Because of the tensile strength decreasing of the friction stir welded wrought magnesium (Mg) alloy compared to the base material, the reasons for the failure of weld has been focused on. After the fracture in transverse tension, the crack went through the welded joint from the center of the weld to the transition zone between the thermal-mechanical affected zone and weld zone. In the present study, the microstructure characteristics and its effect on the facture in the triple junction region is investigated. Based on the metallography and the electron back-scattered diffraction (EBSD) technology, it was observed that a twin band extended from the triple junction region to the middle of weld. The profuse twinning in the twin band was considered to play an important role on the crack propagation from the stir zone edge to the crown zone.

## 1. Introduction

As one of the lightest structural materials, magnesium (Mg) alloys have wide application in the aerospace and automotive industries for the purpose of weight saving [1]. However, the hexagonal close packed structure is a negative factor for the formability of Mg alloys [2]. As a solid-state joining technology, the friction stir welding (FSW) is useful for improving the joining of Mg alloys [3,4]. FSW has been successfully applicated in several Mg alloys systems, such as AZ (Mg–Al–Zn) and AM (Mg–Al–Mn) [5,6]. However, the FSW joints of wrought Mg alloys suffers usually from a problem of tensile strength decreasing, compared to basal metal [7,8,9,10]. Plentiful research on the failure of weld indicated that the crack went through the specific regions, i.e., the transition zone (TZ), after the fracture [11,12,13]. Therefore, the reasons for failure occurring in the regions of FSW Mg alloys have been focused on [14,15,16,17,18].

It is known that the crown zone (CZ), stir zone (SZ) and thermal-mechanical affected zone (TMAZ) are formed in the FSW joint of Mg alloys [19]. The triple junction region is the junction of CZ, SZ and TMAZ. The drastic texture variant among them used to be investigated in studying the deformation mechanism of FSW Mg alloy [20,21]. The grain orientation tilted gradually roughly from parallel to welding direction (WD) in SZ center to parallel to transverse direction (TD) in SZ side, which forms the texture orientation favorable for extension twinning and basal slip at transverse tension near the SZ side. Previous studies indicated that these regions of easy to activate the basal slip and extension twinning can play an important role on the fracture, ascribed to the drastic strain localization on the SZ side [13,17,20]. In the triple junction region, the texture orientation with the tendency to tilt from CZ side toward SZ side resulted in the inhomogeneous extension twinning behavior and strain localization through the region, which was considered as the fracture factor at transverse tension [11,21]. In addition, it was reported that the compression twinning and double twinning in the middle of bottom side of SZ were responsible for crack nucleation and propagation, due to the strain incompatibility at the twin interface [22,23]. Extensive previous studies focused on the fracture behavior in SZ [14,15,16,17,18,20]. However, it is less mentioned how the crack propagated from SZ to CZ. Based on the present work, the understanding of the fracture behavior seems to be further added.

In this paper, the microstructure in the triple junction region was examined when the specimen was stretched along the transverse direction to the stress of 90% ultimate tensile strength (UTS). It was observed that a twin band extended from the triple junction region to the middle of weld. The potential effect on the crack propagation was discussed based on the fracture morphology, metallography observation and electron back-scattered diffraction (EBSD) analysis.

## 2. Materials and Methods

**Figure 1:**
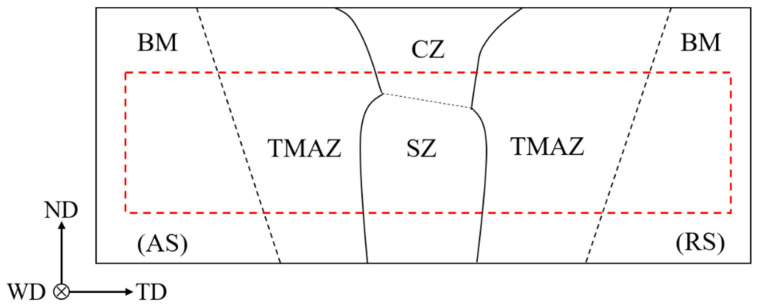
Illustration of the cross-section of friction stir welding (FSW) joint divided into several zones. retreating side (RS) and advancing stand (AS) stand for retreating side and advancing side, respectively. Base materials (BM), thermal-mechanical affected zone (TMAZ), crown zone (CZ) and stir zone (SZ) stand for base material, thermal-mechanical affected zone, crown zone and stir zone, respectively. Normal direction (ND), welding direction (WD) and transverse direction (TD) stand for normal direction, welding direction and transverse direction, respectively. The red dotted rectangle shows the sample position, and the thickness and tensile direction of samples were parallel to the ND and TD of FSW joint, respectively.

Hot-rolled commercial AZ31 Mg alloy (Mg-3%Al-1%Zn) plates were used as the base materials. The thickness of the welded plates was 6 mm. A cylindrical thread pin tool with a probe length of 5.7 mm, a pin diameter of 5 mm and a shoulder diameter of 15 mm was used in the FSW. The welding was conducted with the tool tilt angle of 2.5° at a rotation rate of 1600 rev min^−1^ and a welding speed of 600 mm min^−1^. Dog bone shaped specimens with nominal gage dimensions of 15 mm × 4 mm × 3 mm were prepared for transverse tensile tests. Tensile specimens were cut by the wire-electrode cutting. The thickness and tensile direction of samples were parallel to the normal direction (ND) and transverse direction (TD) of FSW joint, respectively, shown in Figure 1. It is seen that the specimen includes the TMAZ, CZ, SZ and their triple junction region in Figure 1. In addition, tensile samples were machined at least 0.8 mm away from the bottom of the FSW plates, to exclude the possible effect of unwelded layers on the mechanical tests. A tensile test was performed at a strain a rate of 1 × 10^−3^ s^−1^ at room temperature.

The cross-section of the joint vertical to the WD was chosen for microstructure characteristics, via optical microscope (OM, Zeiss, Germany) and EBSD. The samples for OM were etched with a solution consisted of 2 mL distilled water, 2 mL glacial acetic acid, 14 mL ethanol and 0.84 g picric acid. The samples for EBSD analysis were polished at 20 V in a commercial polishing solution AC2 at 20 °C. The EBSD step size was 1.5 μm.

## 3. Results

### 3.1. Undeformed Microstructure

**Figure 2:**
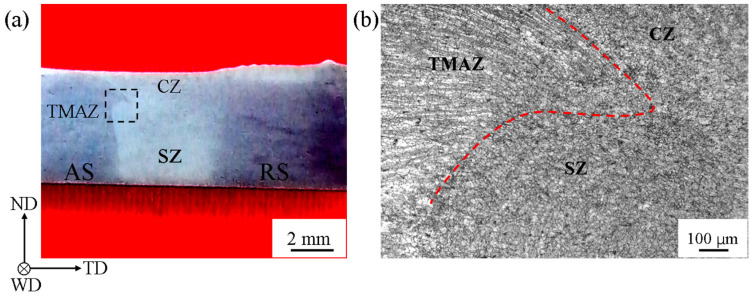
(**a**) Macrograph of the cross-section of FSW joint and (**b**) micrograph of the triple junction region marked by a black dotted rectangle in (**a**).

Figure 2a shows the macrograph of the cross-section of FSW sample after etching. The interface between TMAZ and weld zone (WZ) at advancing side (AS) was manifested. In particular, the triple junction region was marked out with the black dotted rectangle. A trace of sharp corner appeared in the region. Figure 2b shows the micrograph of the triple junction region. The red dotted line roughly outlines the sharp corner in the region. The region was considered to play an important part on the deformation and fracture [24].

**Figure 3:**
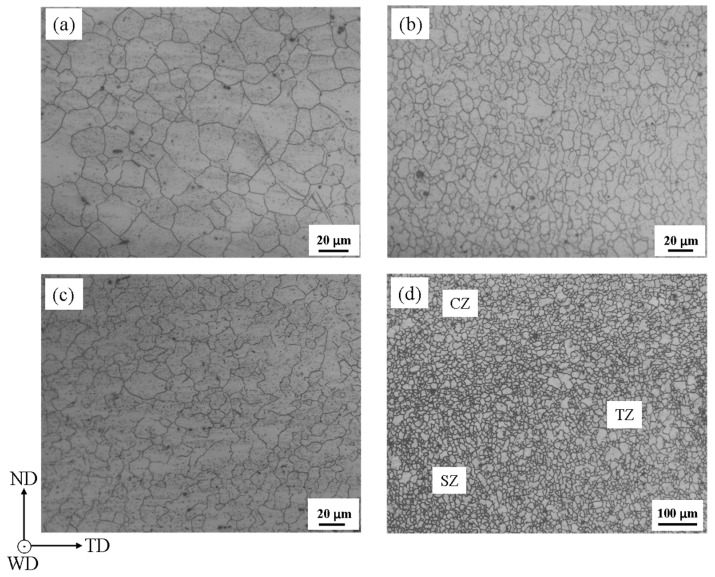
The metallographic photos in TMAZ (**a**), SZ (**b**), CZ (**c**) and the triple junction region (**d**) of undeformed sample.

Because of friction heat and stress from the stir pin during FSW, different microstructures were generated in CZ, SZ, TMAZ and the triple junction region. The initial microstructures in these zones are shown in Figure 3. The mean grain sizes were measured to be 19.32 μm, 8.53 μm and 11.92 μm, in TMAZ, SZ and CZ, respectively, by linear intercept method. It is observed that the grains are roughly uniform in any zone and twins do not appear in the grains before deformation. The grains in both SZ and CZ show the characteristic of the equiaxed crystal, being attributed to the dynamic recrystallization, which resulted in there being few small angle grain boundaries in the grains. The reasons for the bigger grains in CZ than in SZ are related to the more heat caused by the friction between the pin shoulder and base material. In Figure 3d, the grains were separately generated in CZ, SZ and TZ. It is observed that the sizes of grains in the three zones are different with each other. The mean grain size in TZ was measured to be 12.1 μm, by linear intercept method, in Figure 3d. The grains are smaller than that in Figure 3a, implying that the region may be not called simply as TMAZ. Apart from the grain size, it seems that there is not a clear boundary among the three zones in Figure 3d.

**Figure 4:**
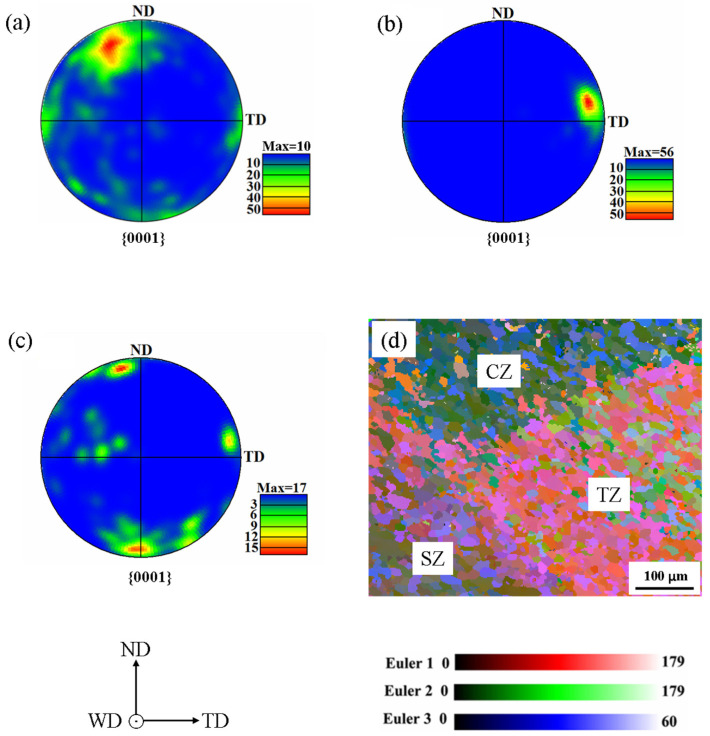
Electron back-scattered diffraction (EBSD) maps: The pole figures in TMAZ close to SZ (**a**), SZ side (**b**) and CZ close to the center of weld (**c**); and (**d**) The Euler map in the triple junction region.

Figure 4a–c show the pole figures of TMAZ close to SZ, SZ side and CZ close to the center of weld, respectively. The texture orientation in TMAZ close to SZ is close to that in base material, which has the texture of a rolled plate. The texture orientations in SZ side and CZ close to the center of weld are roughly parallel to TD and ND, respectively. The texture orientations in the three zones are expected. The similar results were reported in previous studies [19]. The texture orientation of CZ in Figure 4c is favorable for the compression twinning, due to the compressed *c*-axis and the prism slip under enough transverse tension stress. The pole figures of SZ side and TMAZ close to SZ indicate that the extension twins are easy to be activated in the grains, due to the extended c-axis in tension. In Figure 4d, the regions with different Euler colors indicate the distinction of grain orientation among CZ, SZ and TZ. The visual difference demonstrates that the texture orientations among CZ, SZ and TZ are different in the triple junction region. Similar results were reported [11,21,24], and it was validated that the grain orientations in CZ side and SZ side were preference of the basal slip and extension twinning in the triple junction region, respectively, in transverse tension [11,25].

### 3.2. Mechanical Properties and Deformation Behavior

**Figure 5:**
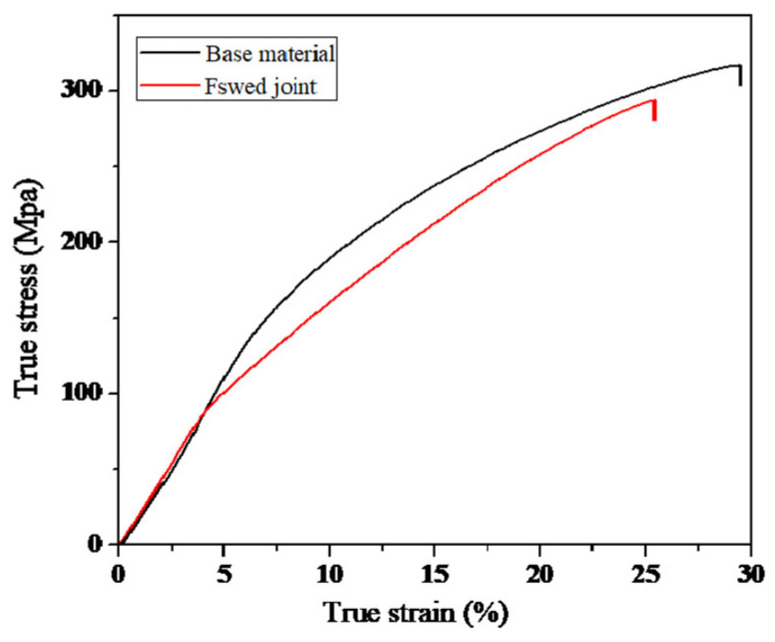
The true stress-strain curve.

Figure 5 shows the true stress-strain curves of the tensile test specimen. The yield strength (YS) and UTS of base material are 113 ± 3.5 MPa and 317 ± 8.1 MPa, respectively, and the elongation is 29 ± 1.4%. The YS and UTS of FSW joint are 81 ± 3.2 MPa and 295 ± 6.1 MPa, respectively, and the elongation is 25 ± 1.2%. The mechanical properties of FSW joint are lower than that of base material. The decreasing of mechanical properties can be described to the texture of SZ side, favorable for extension twinning and basal slip [3,15]. Based on the mechanical properties, the tensile tests were terminated when the stress level was up to 90% of UTS, and then the specimens were prepared for the microstructure examination.

**Figure 6:**
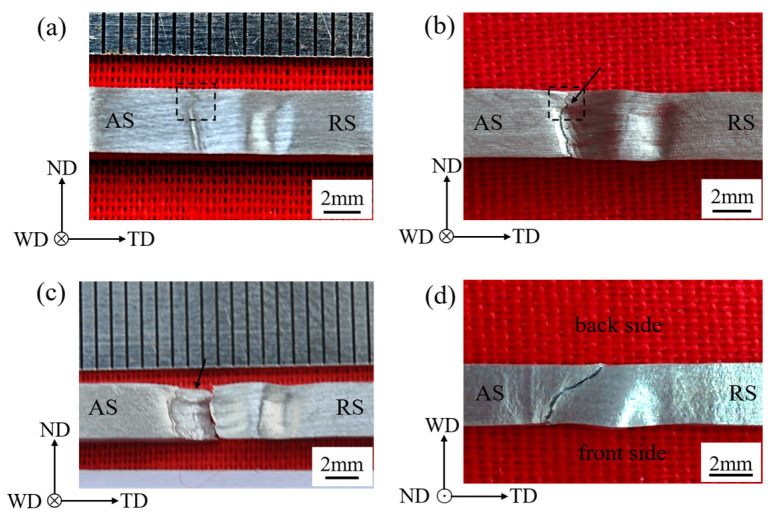
Photographs: (**a**) at 90% ultimate tensile strength (UTS), (**b**,**c**) after fracture on the front side of FSW samples, (**d**) after fracture on the top side of FSW samples. The triple junction region is marked with the black dotted rectangle in (**a**,**b**).

Figure 6a shows that the ‘concave-convex’ appearance on the front side of weld was at 90% UTS. The deformation relief appeared at AS. The deformation relief was at the interface between TMAZ and WZ, ascribed to the drastic texture variation at two sides of the interface [20,21]. In particular, a deformation relief with a sharp corner appeared in the region marked out with the black dotted rectangle. The deformation feature was also observed in the bending test of the FSW Mg alloy [12]. Previous studies indicated that the deformation relief with sharp corner is in the triple junction region [11]. The photograph on the front side of FSW specimen after fracture is presented in Figure 6b. It is obvious that the crack propagated along the deformation relief in Figure 6a. The feature of crack propagation was also reported in other papers [8,13]. It was considered that the microstructure and texture orientation favorable for the extension twinning and basal slip near the interface played an impact on the fracture [11,20]. In Figure 6b, it was noted that the crack corner pointed out by a black arrow appeared in the triple junction region, and it was related to the deformation relief with sharp corner in Figure 6a. The photograph of the fracture surface of sample was shown in Figure 6c. It is seen that a boundary originated from the crack corner and divided the fracture surface into upper and lower parts. The fracture surface above the boundary was inclined backward, as pointed out by the black arrow, and was mainly in CZ (the triple junction region was at the interface between SZ and CZ). The fracture surface below the boundary is mainly in SZ, influenced by the “onion ring structure” [23]. Figure 6d shows that the crack propagated from the center of weld to the WZ side on the WD-TD plane. Based on the crack propagation, the center of weld and the WZ side have been focused on as the important regions for the failure analysis [13,23].

**Figure 7:**
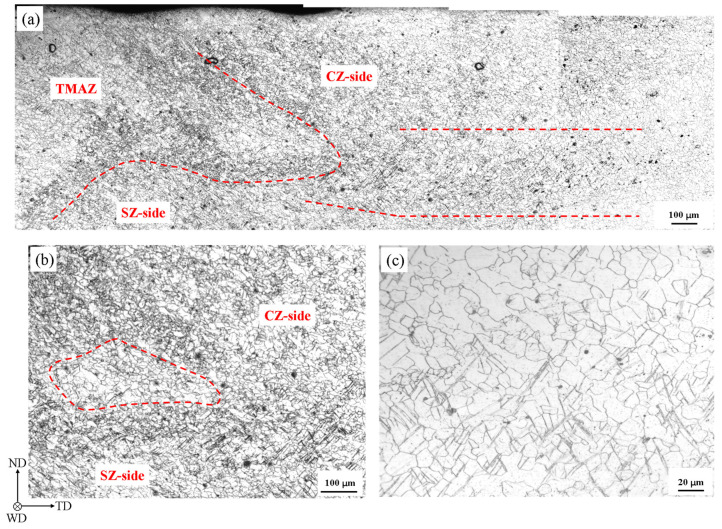
The metallographic photos at 90% UTS. (**a**) The metallograph near the triple junction region. The SZ side (SZ-side), CZ side (CZ-side) and TMAZ were marked with the red words in the relative regions in (**a**,**b**). The twin band was marked between two red dotted lines in (**a**). The partial enlarged details on the left of and in the right end of the twin band were shown in (**b**,**c**), respectively.

Figure 7a shows the microstructure of the triple junction region at the stress level of 90% UTS. More information appeared in the metallograph, compared to Figure 3d. Many twins were generated at SZ side and CZ side. Abundant twin boundaries formed the obscure black boundary, both between TMAZ and CZ and between TMAZ and SZ. The boundary was painted with the red dotted lines, and the outline was related to the sharp corner shown in Figure 2a and Figure 6a. It is worth noting that a twin band was formed, as marked between the red dotted lines in the right side of Figure 7a. Abundant twins were generated in the twin band, whose length was more than 1 mm. The closer the twin band is to the triple junction region, the more twins appeared. In the opposite direction, the twins appear fewer and fewer, and the twins do not nearly appear on the right of the marked zone. The details on the left of the twin band were shown in Figure 7b. It was observed that many twins appeared in SZ side and less twins appeared in CZ side. Besides, a region containing few twinning was marked by the red dotted circle. The inhomogeneous twinning behavior could intensify the strain localization in the triple junction region [11]. Thus, it was inferred that the circled region corresponded to the sharp corner shown in Figure 6a. It was also seen that the twin band originated from the right of the circled region and joined the twins in SZ side in Figure 7a,b. It implies that the influence of strain localization extended to the middle of weld along the twin band. The partial enlarged details in the right end of the twin band are shown in Figure 7c. It is seen that many twins appeared below the figure region. On the contrary, above the figure region, few twins appeared. The twinning was probably inhibited due to the active slip in the position at the stress level.

**Figure 8:**
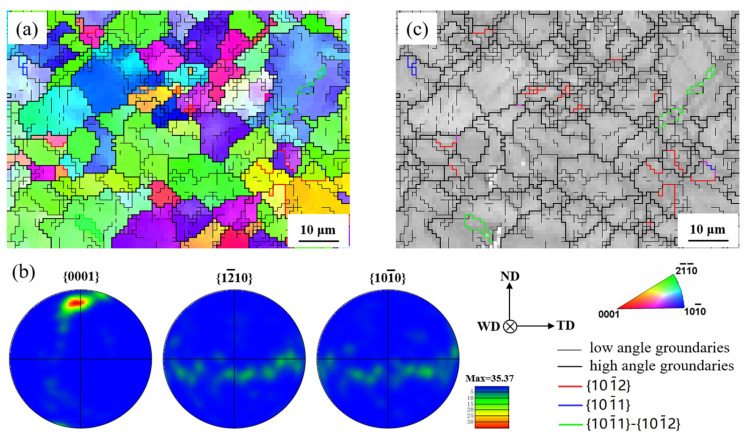
EBSD orientation maps of the micro-region in the right end of twin band: (**a**) IPF map, (**b**) pole figures and (**c**) grain boundary map.

Because the microstructures of samples were examined at the stress level of 90% UTS, the EBSD maps of the twin band close to the triple junction region did not acquire a high enough index rate. Thus, the analyzed region was selected in the right end of the twin band. The inverse pole figure (IPF) of the selected region shows that grains are roughly uniform in Figure 8a, and the relative pole figures indicate that *c*-axes of grains in the region are mainly parallel to the ND in Figure 8b. The texture orientation is in CZ and favorable for the compression twinning and prism slip comparing with that in Figure 4c. The grain boundary map reflects that many low angle grain boundaries were formed in the grains and the {10$\bar{1}$2} extension twinning, {10$\bar{1}$1} compression twinning and {10$\bar{1}$1}–{10$\bar{1}$2} double twinning were generated in the twin band in Figure 8c. Primary {10$\bar{1}$1} twinning is favorable for the secondary {10$\bar{1}$2} twinning during the subsequently transverse tension, due to the lattice rotation about 56° around <1$\bar{2}$10> direction. The texture orientation in the region is not preference of the extension twinning, but many low angle grain boundaries may imply that the extension twinning was induced by the slip, which will be considered in the later research.

## 4. Discussion

As mentioned above, abundant twins were generated in the triple junction region at the stress level of 90% UTS. However, the distribution of twins was inhomogeneous and mainly in the SZ side and the twin band. The inhomogeneous twinning intensified the strain localization in the triple junction region. The strain localization was generally considered to play an important role on the fracture behavior [10,20,26]. Previous studies indicated that it depended on the deeper necking in SZ side; whether the fracture appeared at AS or retreating side (RS) of weld [10,20]. In the current work, the apparent strain localization appeared in SZ side and the triple junction region at AS, seeing the deformation relief shown in Figure 6a. Thus, the crack occurred in these zones, as shown in Figure 6b. Besides, in the twin band, the activated twinning contained the {10$\bar{1}$2} extension twinning, {10$\bar{1}$1} compression twinning and {10$\bar{1}$1}–{10$\bar{1}$2} double twinning. The compression twinning and double twinning were believed to be responsible for the failure of the weld [22]. Mironov et al. observed that the failure of the FSW sample originated from the {10$\bar{1}$1}–{10$\bar{1}$2} double twinning, which were generated in the middle of bottom surface of SZ, at the transverse tension [23]. Furthermore, he thought that the effect of extensive {10$\bar{1}$2} twinning occurred at the SZ edges on the failure attributed to the {0001}<11$\bar{2}$0> orientation change, subsequently boosting the secondary {10$\bar{1}$1} twinning [27]. Therefore, the profuse twinning in the twin band may provide the path for the crack propagation from the SZ edge to CZ, and from the triple junction region to the center of the weld. However, with the decreasing of twinning above the twin band, the effect of twinning on the crack propagation was weakened. On the front side of weld, the crack propagation turn to the left (pointed out by the black arrow in Figure 6b), where the strain localization became intense (seeing the deformation relief in the Figure 6a).

## 5. Conclusions

In summary, the microstructure characteristics and fracture behavior in the triple junction region was studied using the metallography observation and EBSD technique. It was observed that a boundary appeared between CZ and SZ on the fracture surface of weld. At the boundary, a twin band, which was more than 1 mm long, extended from the triple junction region to the middle of the weld at the stress of 90% UTS. The twin band jointed the twins in the SZ side on the left, which intensified the strain localization in the triple junction region, and went into CZ on the right. The twin band contained {10$\bar{1}$2} extension twinning, {10$\bar{1}$1} compression twinning and {10$\bar{1}$1}–{10$\bar{1}$2} double twinning. The profuse twinning in the twin band may provide the path for the crack propagation from the SZ edge to CZ.
